# Soft and tough bio-composites via integration of agricultural products and polymer gels

**DOI:** 10.1080/14686996.2025.2604923

**Published:** 2025-12-18

**Authors:** Honoka Matsuura, Kagari Maruyama, Shou Ohmura, Jian Ping Gong, Tasuku Nakajima

**Affiliations:** a Graduate School of Life Science, Hokkaido University, Sapporo, Japan; b Faculty of Advanced Life Science, Hokkaido University, Sapporo, Japan; c Institute for Chemical Reaction Design and Discovery (WPI-ICReDD), Hokkaido University, Sapporo, Japan

**Keywords:** Composite, hydrogel, bio-composite, reinforcement, eringi, kanpyo

## Abstract

As an extension of fiber-reinforced plastics, research on fiber-reinforced soft hydrogels has attracted remarkable attention. From the perspective of sustainability, it is desirable to use biopolymers such as cellulose and chitin as the fibrous phase of these hydrogel composites. However, obtaining biopolymer-based fibers from plants or fungi generally requires environmentally harmful processes such as extraction, purification, and reconstruction of the target biopolymers. To avoid this problem, this study aimed to obtain tough biopolymer/hydrogel composites with minimal environmental impact. Specifically, minimally processed eringi (king oyster mushroom) and kanpyo (dried shaved gourd) were directly used as the fibrous phase, and hydrogel matrices were prepared within them to make the bio-composites. The hierarchical fibrous structure of the biopolymers inherently present in eringi and kanpyo was well preserved in the bio-composites. The resulting composites exhibited high strength and toughness originating from the well-aligned fibrous biopolymers in the bio-composites.

## IMPACT STATEMENT

Mechanically robust and environmentally friendly bio-composite materials have been developed via a simple process that integrates agricultural products with hierarchical fibrous structures directly into hydrogel matrices, without any complex pretreatment.

## GRAPHICAL ABSTRACT

**Figure:**
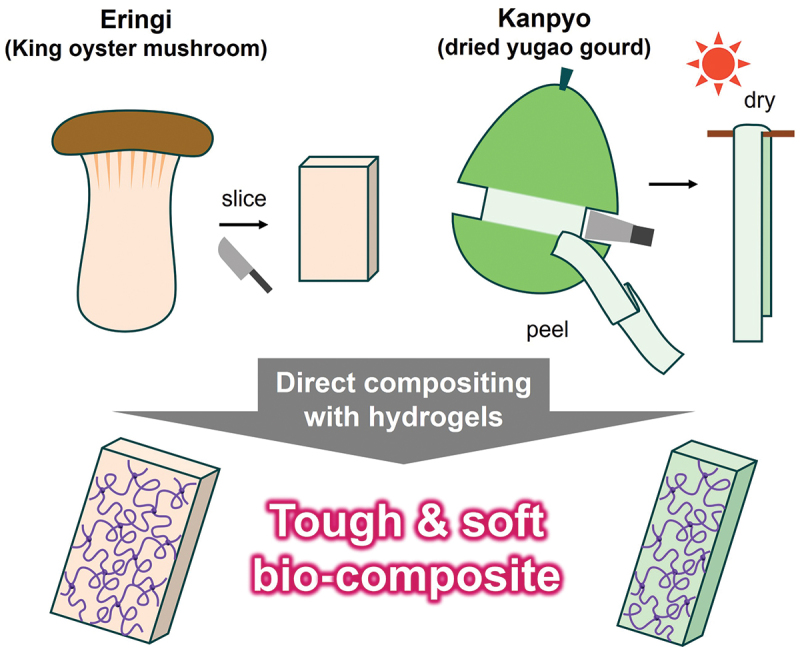

## 1. Introduction

Biological tissues typically exhibit highly anisotropic and hierarchical architectures, which play key roles in their physiological functions, such as directional material transport (e.g. vascular bundles), anisotropic motion (e.g. muscle fibers), and mechanical linkage between hard tissues (e.g. tendons). As an example of the anisotropic hierarchical structure, vascular bundles of plants are composed of anisotropic cells arranged in multiple layers, with each cell wall mainly constructed from cellulose microfibrils [1]. This architecture is not only essential for specialized biological functions but also significantly enhances the mechanical toughness. For instance, the alignment of vascular bundles in plants enables efficient unidirectional molecular transport while facilitating mechanical stress distribution and energy dissipation across multiple scales [1–3]. These fibrous arrangements optimize mechanical performance by balancing strength and flexibility, allowing organisms to resist fracture under dynamic loads.

Similarly, the integration of rigid long-fiber structures into artificial materials is widely known as an effective strategy to improve their toughness [4]. Embedding stress-bearing rigid fibers within a less rigid matrix often leads to increased fracture stress and enhanced fracture resistance. While resin matrices are typically used for fiber-reinforced composites, this approach can also be applied to soft elastomeric matrices [5–10]. Radial tires are reinforced with layered steel or nylon cords, and super-tough hydrogels have been developed by embedding steel, glass, or carbon fibers in gels. For reinforcing artificial materials, synthetic fibers are preferably used due to their excellent strength, stability, and durability. However, three major challenges are associated with the use of synthetic fibers in tough composites. First, widely used synthetic fibers such as glass and carbon are non-biodegradable and difficult to recycle, posing environmental concerns. Second, the energy required to produce these fibers from raw materials is substantial. Third, fiber orientation in synthetic composites tends to be simplistic and lacks the hierarchical complexity found in natural tissues.

Therefore, researchers have explored biopolymer-based fibers such as chitin and cellulose fibers as substitutes for synthetic ones for composites [11–13]. While their biodegradability offers environmental advantages, the other two challenges, which are energy-intensive processing and loss of hierarchical structure, remain only partially resolved. Producing biopolymer fibers typically involves multiple steps [14–17], including extraction, purification, and structure reconstruction of the target biopolymers from native biological tissues, which consume significant amount of time and energy. In certain procedures, the use of hazardous reagents such as strong acids/bases and organic solvents are necessary. Furthermore, such processing destroys the native higher-order architecture of biological tissues, resulting in simplified reconstructed structures.

To tackle this issue, we have proposed an alternative strategy: bio-composites fabricated using a fibrous biological tissue as is with minimal processing [18]. As an example, some of the authors demonstrated that squid mantle embedded within a polyacrylamide (PAAm) matrix produced the bio-composite hydrogels with high fracture resistance [18]. These composites were simply prepared by immersion of squid in the gel precursor and synthesizing the gel network inside the squid. The highly oriented muscle fibers in squid effectively reinforced a crack front to suppress its propagation, markedly enhancing toughness of the composites. However, given the economically valuable nature of squid as a food and its unsustainable supply, use of squid as a structural material is impractical.

**Figure 1.**
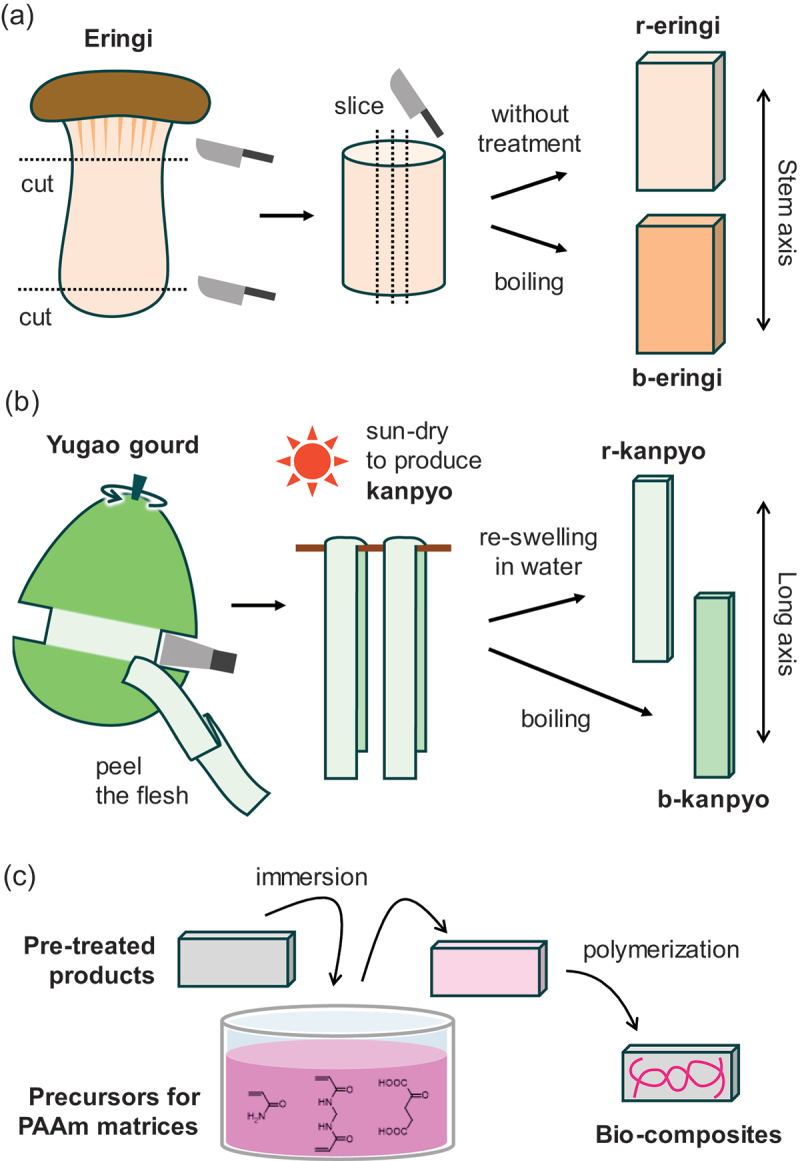
(a, b) Pre-treatment processes of agricultural products for the bio-composites; (a) eringi and (b) kanpyo. (c) The method to prepare the bio-composites using polyacrylamide as a matrix.

Here we report on the fabrication of bio-composites using fibrous agricultural products. Compared to marine-derived materials such as squid, agricultural products are more sustainable, making them more suitable candidates for material development. Ideal agricultural products for bio-composite production should be inexpensive, available year-round, and sufficiently large to serve as composite substrates for real-world use. We selected eringi mushrooms (*Pleurotus eryngii*) and kanpyo (dried gourd strips), which are easily found in Japanese local markets. An eryngi mushroom, also called king oyster mushroom in some countries, is an edible mushroom, notable for its large and thick stem ( = stipe) (Figure 1(a)). An eringi is an industrially cultivatable mushroom, and it is constantly supplied to markets throughout the year. Especially in Japan, eringi mushrooms with long and thick stem are preferred and cultivated accordingly to meet this demand. A stem of eringi contains hierarchical and anisotropic structure of biopolymers. The stem is made up of anisotropic hyphae aligned along the stem axis. The hyphae are covered by cellular walls, which are composed of highly crystalline chitin microfibrils embedded within less oriented matrices primarily composed of β-glucan [19–21]. Kanpyo is a Japanese traditional dried vegetable made by shaving the inner flesh of yugao gourds (*Lagenaria siceraria*) into long strips and hanging them for sun-drying (Figure 1(b)) [22]. The flesh of yugao gourds consists of ~95 wt% water and ~5% residue, the majority of which would be cellulose, with small amounts of pentosan and lignin [23]. Kanpyo is used for maki-sushi and other dishes after being rehydrated (and typically simmered in sweet soy sauce) [24]. While a raw gourd has a crisp texture without fibrousness, the rehydrated kanpyo is characterized by its chewy and fibrous texture. This change of texture can be attributed to the collapse of cellular walls during the dehydration process, which is commonly observed during the drying of vegetables [25]. Since kanpyo is a traditional preserved food that maintains quality for up to one year, a constant quality of kanpyo is supplied throughout the year.

In this paper, we aim to synthesize soft matrices inside the minimally treated eringi and kanpyo to obtain tough bio-composites based on the objective of utilizing the higher-order structure of these products and obtaining composites with low energy input. Since inside of agricultural products is filled with water at least partially, hydrogels are good candidates for making bio-composites because of their potential high chemical affinity to agricultural products. We first selected a polyacrylamide (PAAm) hydrogel, which is a common hydrogel having hydrogen bonding capability. We also tried gelatin hydrogels as a bio-based soft matrix selectively suitable for raw eringi. Fibers in vegetables and mushrooms typically undergo hydration upon boiling, which enhances their compatibility with water and other hydrophilic molecules. To investigate the effect of this hydration on the composite mechanical properties, we utilized two states of eringi and kanpyo: the raw state and the state subjected to minimal boiling treatment to induce their hydration (eringi at 65°C and kanpyo at 95 °C) [26,27].

## 2. Experiments

### 2.1. Materials

Factory-produced eringi mushrooms with thick and sturdy stems (Yukiguni eringi, Yukiguni Factory Co., Ltd., Japan) were immediately used after delivery. Sun-dried kanpyo strips without bleaching treatment (Sokensha Co., Ltd., Japan) were used as received. *N,N*’-methylene(bis)acrylamide (MBAA), ammonium persulfate (APS), and gelatin were purchased from Fujifilm Wako Pure Chemical Co., Japan and used as received. Acrylamide (AAm) was purchased from Junsei Chemical Co., Ltd., Japan and used without further purification.

### 2.2. Pre-treatment of eringi and kanpyo

The pre-treatment processes of eringi and kanpyo are schematically shown in Figure 1(a,b). The eringi mushrooms were sliced to a thickness of around 2 mm along their stem axis after removal of the caps. A sharp razor blade was used for the cutting to minimize damage to eringi microstructure during slicing. To avoid variations in physical properties originating from the sample position in the mushroom, the slices containing the center of the stem were selectively used. Some of the sliced eringi were boiled in 65°C water for 5 minutes and then immediately cooled in 25°C water. The raw and boiled eringi slices are referred to as r-eringi and b-eringi, respectively. The dried kanpyo strips were first rinsed with pure water to remove possible dust. Some of the rinsed kanpyo strips were just immersed in 25°C water for 1 day, and others were boiled in 95°C water for 7 minutes and then immediately cooled in 25°C water for 1 day. The former and the latter are referred to as the rehydrated kanpyo (r-kanpyo) and boiled kanpyo (b-kanpyo) strips, respectively.

### 2.3. Preparation of bio-composites

#### 2.3.1. Bio-composites with PAAm matrix

To prepare the precursor solutions, 2, 4, and 6 M of AAm, 0.1 mol% of MBAA, and 0.1 mol% of APS were dissolved in pure water, where mol% represents the relative molar concentration with respect to the AAm concentration. The r-/b-eringi or r-/b-kanpyo were immersed in the excess precursor solutions for at least 1 day at 4°C. The eringi and kanpyo were sandwiched between two glass plates, and the surrounding space was filled with the precursor solution. The assembly was heated at 60°C for 10 hours for polymerization of AAm to obtain the bio-composites with PAAm matrix. The obtained bio-composites are called eringi/PAAm-*C* or kanpyo/PAAm-*C* composites, where *C* = 2, 4, or 6 is the AAm concentration (M) of the matrix precursor solution.

#### 2.3.2. Bio-composites with gelatin matrix

The r-eringi were immersed in 20 or 30 wt% of gelatin aqueous solutions at 60°C for 1 day. The eringi were then sandwiched with two glass plates and placed at 4°C for 1 day to obtain the bio-composites with gelatin. The obtained bio-composites are called r-eringi/gelatin-*C* composites, where *C* = 20 or 30 is the gelatin concentration (wt%) of the precursor solution.

### 2.4. Microscopy observation

The fibrous structures of eringi and kanpyo were examined using optical microscopy (OM) and scanning electron microscopy (SEM). For optical microscope observation, the *r*- and b-eringi were torn along the stem axis and sliced using a razor blade. The *r*- and b-kanpyo were also sliced in the same way along the long axis. These slices were mounted on slides and observed using a Nikon Eclipse LV100POL (Nikon Co., Japan) in transmission mode. SEM imaging was conducted under low vacuum conditions using a JSM-6010LA (JEOL Ltd., Japan) on the same samples. The cut samples were observed directly without any pretreatment, such as drying or ion sputtering, using an accelerating voltage of 10 kV under low vacuum.

### 2.5. Weight fraction measurement

Weight fractions of the contents in the materials were analyzed as shown below. The solid weight fraction of the eringi and kanpyo, *w*_a_, was calculated as *w*_a_ = *m*_d_/*m*_a_, where *m*_d_ and *m*_a_ are the weights of the sample at the completely dried state and initial wet state, respectively. Subsequently, the bio-composites were prepared from the (pre-treated) agricultural products with a known weight, *m*_a_. Using the weight of the wet agricultural product, *m*_a_, its solid weight fraction, *w*_a_, and the weight of the composite at its wet state, *m*_c_, the weight fraction of the product-derived solid component within the composite, *w*_p_, was calculated as

(1) $$w_{p}=\frac{m_{a}w_{a}}{m_{c}}.$$

### 2.6. Mechanical testing

The mechanical tests were performed with a commercial tensile tester (Instron 5965, Instron Co., U.S.A.) at 25°C under ambient humidity conditions. For the uniaxial tensile test, the samples were cut into dumbbell shape standardized as JIS K6251-7 (gauge length 12 mm, width 2 mm). For the samples derived from eringi, samples were cut such that their longitudinal axis was either perpendicular or parallel to the stem axis of the eringi. The specimen was uniaxially stretched until its rupture with a constant velocity of 100 mm/min. This velocity is considered high enough to ignore drying during testing. The nominal stress, *σ*, was defined as the tensile force divided by the initial cross-sectional area of the specimen. The deformation ratio, *λ*, was defined as the length of the specimen divided by its initial gauge length. The strain, *ε*, was defined as *λ* −1. The fracture stress and the strain at break were defined as *σ* and *ε* at the failure of the specimen, respectively.

Fracture tests were conducted using a single-edge notch tensile test configuration. Each sample was cut into a rectangular shape (20 mm in length and 7 mm in width) with a 2 mm pre-notch introduced at the center of the long edge. Uniaxial deformation was applied to the specimen along its longitudinal direction at a constant velocity of 60 mm/min at 25°C under ambient humidity conditions. The fracture energy, *G*, was calculated using the following equation:

(2) $$G=\frac{6W_{b}c}{\sqrt{\lambda_{b}}},$$

where *λ*_b_ denotes the onset deformation ratio of crack propagation of the notched specimen, *W*_b_ represents the area under the *σ*–*λ* curve the unnotched specimen up to *λ*_b_, and *c* = 2 mm is length of the pre-notch.

## 3. Results

### 3.1. Structure evaluation of eringi, kanpyo, and their bio-composites

**Figure 2.**
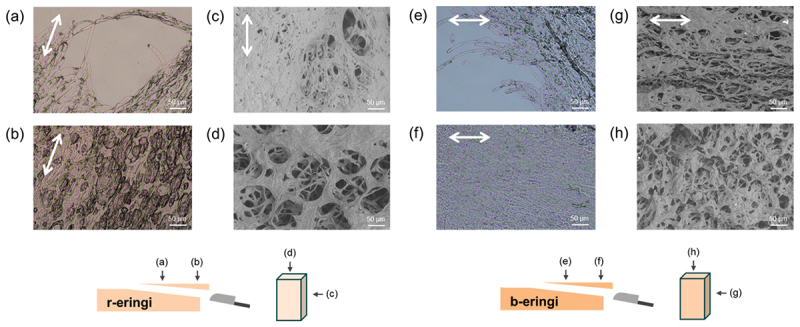
Microscope images of eringi. (a, b) Optical microscope (OM) images of the (a) thin and (b) thick slices of the r-eringi. (c, d) Scanning electron microscope (SEM) images of the r-eringi; (c) cross-sectional and (d) longitudinal-sectional views. (e, f) OM images of the (e) thin and (f) thick slices of the b-eringi. (g, h) SEM images of the b-eringi; (g) cross-sectional and (h) longitudinal-sectional views. All the SEM images were captured in low-vacuum mode. White arrows indicate the stem axis.

The structures of eringi and kanpyo were analyzed. The raw eringi (r-eringi) exhibited a fibrous surface texture. Figure 2(a,b) shows the optical microscope (OM) images of the thin and thick slices of the r-eringi. In the image of the thin slice, anisotropic fungus hyphae oriented along the stem axis with a diameter of ~10 µm were observed. In the image of the thick slice, anisotropic bubbles (voids) with a diameter of ~100 µm were observed. These hierarchical structures were also found in the scanning electron microscope (SEM) images. The cross-sectional SEM image of r-eringi (Figure 2(c)) revealed 100 µm-scale pores, corresponding to the voids, and ~10 µm-scale fibers, corresponding to the single hypha. In the longitudinal-sectional view (Figure 2(d)), fibers loosely aligned along the stem axis were observed. Upon boiling at 65°C, the surface of the eringi became smoother with less fibrous texture. The hyphae were still observed in the optical microscope images, but the large pores were not found (Figure 2(e,f)). Upon SEM observation, smaller pores approximately 10 µm in diameter were observed instead of the large pores, and the alignment of the hyphae was less obvious (Figure 2(g,h)). These results indicate that eringi originally has a hierarchical anisotropy consisting of air-filled pores, anisotropic fungus hyphae, and the molecules consisting of the hyphae, and boiling causes the pores to disappear. Regarding this finding, the solid weight fractions of *r*- and b-eringi were measured as 0.331 and 0.114. The lower solid weight content in b-eringi is due to the replacement of air in the pores with water.

**Figure 3.**
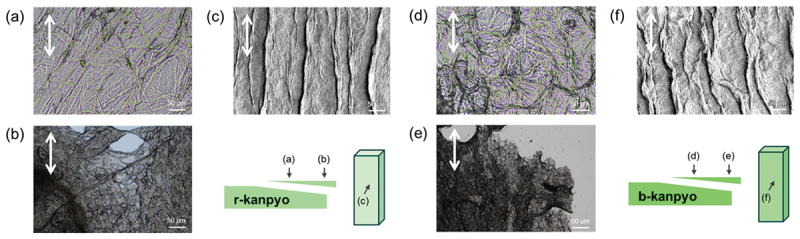
Microscope images of kanpyo. (a, b) OM images of the (a) thin and (b) thick slices of the r-kanpyo. (c) SEM images of surface of the r-kanpyo. (d, e) OM images of the (d) thin and (e) thick slices of the b-kanpyo. (f) SEM images of surface of the b-kanpyo. All the SEM images were captured in low-vacuum mode. White arrows indicate the long axis.

For preparation of kanpyo, peeled gourd flesh strips are hung vertically and dried, which likely causes the fibrous aggregations aligned along the longitudinal axis of the strip. Figure 3(a,b) shows the OM images of the thin and thick slices of the rehydrated (r-)kanpyo. The pores around 100 µm and fibrous structures around 1 µm were observed in the thick and thin samples, respectively, suggesting a hierarchical fibrous structure in kanpyo. While anisotropy was not clearly observed in the OM images, the SEM image (Figure 3(c)) revealed the fibrous structures aligned with the long axis of the kanpyo. Figure 3(d-f) shows the OM and SEM images of the b-kanpyo. The OM images revealed that the fibers appeared less straight than those in r-kanpyo. This structural change is likely attributable to the loosening of intermolecular interactions (mainly hydrogen bonds) induced by heating. On the other hand, no clear differences were observed in the SEM images, suggesting that 7-minute boiling of kanpyo did not destroy their fundamental anisotropic structure. The solid weight fractions of r- and b-kanpyo were measured as 0.549 and 0.150. The reduction of the solid weight content after boiling would be attributed to rehydration and swelling of kanpyo.

**Figure 4.**
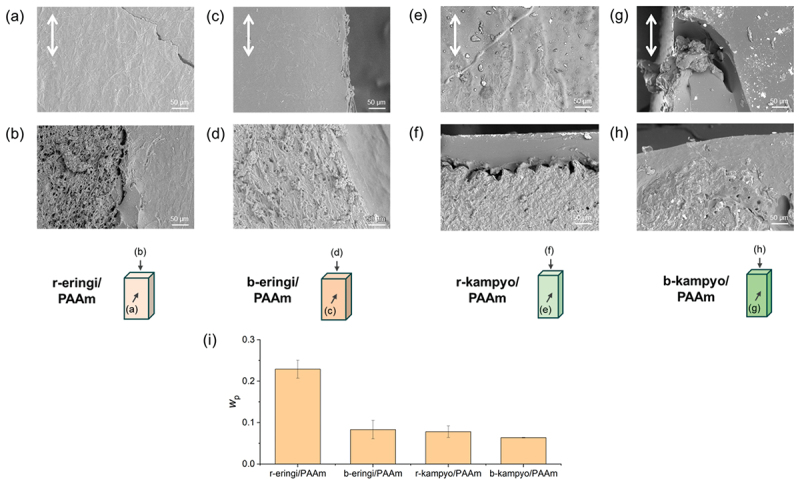
(a−h) SEM images of the (a,b) r-eringi/paam-6, (c,d) b-eringi/paam-6, (e,f) r-kampyo/paam-6, and (g,h) b-kampyo/paam-6 composites; (a,c,e,g) surface views and (b,d,f,h) cross-sectional views. All the SEM images were captured in low-vacuum mode. White arrows indicate the stem axis (eringi) or long axis (kanpyo). (i) Weight fraction of the solid originating from the agricultural products in the composites, *w*_p_.

Subsequently, eringi- and kanpyo-based bio-composites with PAAm matrix have been synthesized as shown in Figure 1(c). The AAm concentration in the matrix precursor solution was first fixed at 6 M. Figure 4 shows the SEM images of the bio-composites. In all cases, the surface images exhibited a smooth and filled structure, while fibrous structures were embedded in the materials. Cross-sectional views revealed distinct fibrous morphologies, with size scales comparable to those of the original agricultural products. These results indicate that the PAAm matrices were incorporated into the eringi and kanpyo while maintaining their original hierarchical structure. Observation of the cross-sections revealed that pores were still visible in the r-eringi/PAAm composite, whereas the pores were not observed in the other composites. To gain deeper insight into this point, the weight fraction of the product-derived solid component, *w*_p_, was measured for each composite. Figure 4(i) shows that *w*_p_ of the r-eringi/PAAm composite was large at 0.23, while that for the other three composites was in the range of 0.06 to 0.08. From these data, it is considered that the amount of PAAm matrix introduced was insufficient in the r-eringi/PAAm composite, resulting in the partial retention of gas-filled pores. In contrast, the PAAm matrix was fully introduced into the interior of the other composites.

### 3.2. Mechanical properties of eringi and kanpyo/polyacrylamide bio-composites

**Figure 5.**
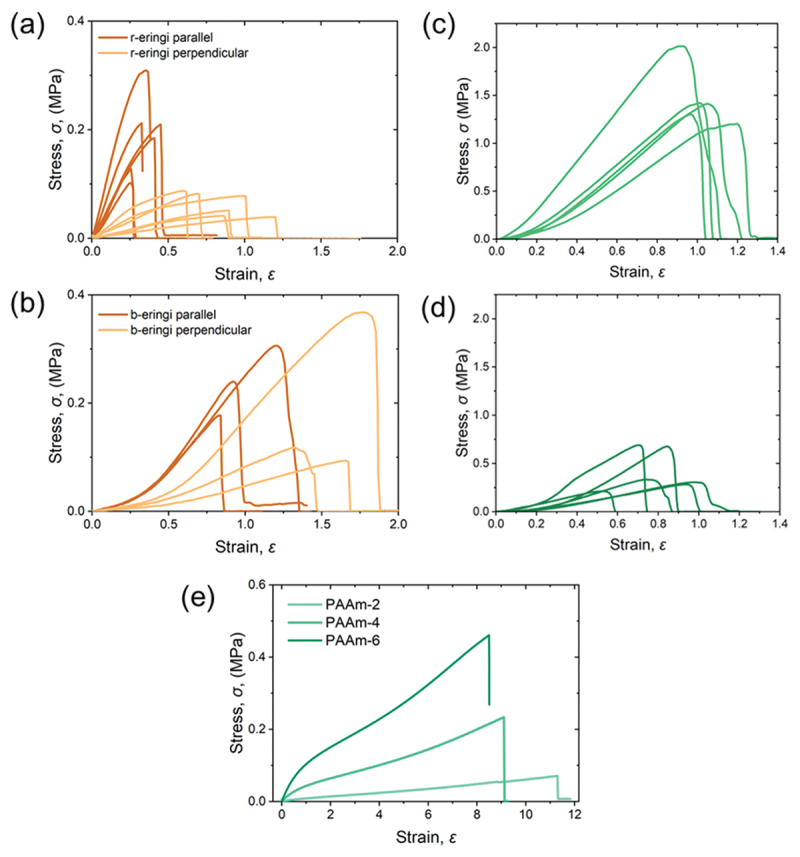
Uniaxial tensile stress-strain curves of the (a) r-eringi, (b) b-eringi, (c) r-kanpyo, (d) b-kanpyo, and (e) PAAm-*C* hydrogels.

Uniaxial tensile tests were conducted on the obtained samples. Figure 5(a-d) presents the uniaxial stress-strain curves of pure eringi and kanpyo without PAAm matrices. In all cases, the shape of the stress – strain curves varied significantly between specimens. This would be attributed to the individual differences commonly observed in bio-derived samples. As shown in Figure 5(a), the r-eringi exhibited significant mechanical anisotropy. Stretching along the stem axis led to higher moduli and fracture stresses, whereas stretching perpendicular to the axis resulted in larger strain at break. This behavior is attributed to the presence of hierarchical fibrous structures in eringi, which resist deformation along the stem axis. On the other hand, b-eringi exhibited less mechanical anisotropy, and its strains at break were larger than those of the r-eringi regardless of the stretching direction (Figure 5(b)). This is consistent with the previous report about compressive properties of eringi [28], and likely due to disruption of the hierarchical structure of the eringi caused by boiling, possibly attributed to partial dissociation of hydrogen bonds and hydration. In the case of kanpyo, r-kanpyo demonstrated superior Young’s moduli and fracture stresses compared to b-kanpyo. It has been known that heating of kanpyo reduces its strength [27], which is also considered to result from partial breakdown of hydrogen bonding during heating, which disrupts the higher-order structure and leads hydration. While long-boiled kanpyo is preferred for food, unboiled (just rehydrated) kanpyo is superior from a materials strength perspective. When comparing eringi and kanpyo, kanpyo exhibited remarkably larger fracture stress. Origin of the difference in strength between them is that the kanpyo underwent drying process but eringi did not. Drying of biopolymers typically promotes their aggregation and the formation of robust fiber structures, which tends to lead to higher modulus and strength [25]. Figure 5(e) shows the representable stress-strain curves for the PAAm-*C* matrices alone, where *C* is 2, 4, and 6 (M). The PAAm hydrogels exhibit lower modulus and larger extensibility than both eringi and kanpyo, indicating their suitability as a soft and stretchable matrix. The fracture stress of the PAAm matrices increased with their concentration. The PAAm-6 matrix exhibited the highest fracture stress among the tested samples, while the value remained around 0.5 MPa.

**Figure 6.**
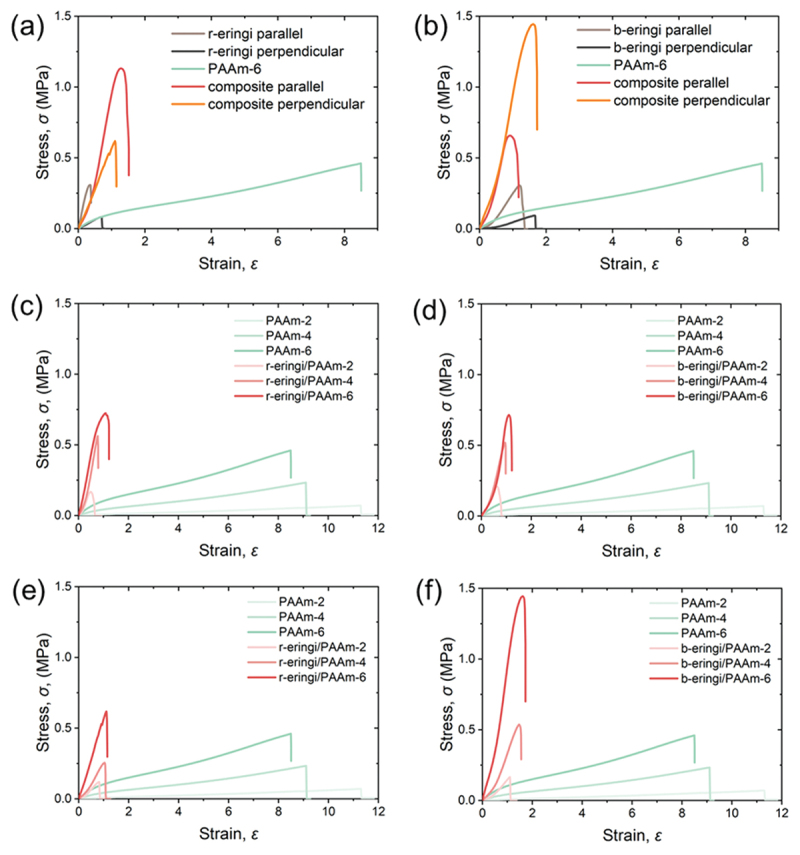
(a,b) Tensile stress-strain curves of the eringi/paam-6 bio-composites and their parents; (a) r-eringi, (b) b-eringi. (c-f) tensile stress-strain curves of the eringi/PAAm-*C* bio-composites and corresponding PAAm-*C* matrices; (c) r-eringi, parallel, (d) b-eringi, parallel, (e) r-eringi, perpendicular, (f) b-eringi, perpendicular.

Subsequently, uniaxial tensile test results of the eringi and kanpyo/PAAm bio-composites are reported. Figure 6(a,b) shows the stress-strain curves of the r/b-eringi, PAAm-6, and r/b-eringi/PAAm-6 bio-composites stretched parallel or perpendicular to their stem axes. By integrating a stiff yet brittle eringi with a stretchy and relatively strong PAAm matrix, we successfully developed bio-composites that exhibit both high stiffness and strength. When stretched parallel to the axes, the composites exhibited large elastic moduli originating from the fibrous structure of eringi. On the other hand, the fracture stresses of the composites were remarkably improved in comparison with those of eringi. In the case of r-eringi/PAAm-6 composite, its fracture stress was even greater than the sum of the fracture stress of the two components. When stretched perpendicularly, both moduli and fracture stresses were much improved in comparison with those of eringi. While the eringi/PAAm composites exhibited high fracture stress regardless of the presence or absence of a boiling process, the b-eringi/PAAm-6 composite stretched perpendicularly exhibited the largest fracture stress. This could be a result of the pore elimination by boiling, which permitted a larger amount of PAAm to be introduced. It is analogous to the behavior of the stiff composites showing decrease of strength when pores are retained [29].

Figure 6(c–f) shows the stress – strain curves of the r/b-eringi/PAAm-*C* composites, where *C* is 2, 4, and 6 M. Interestingly, the strength of the composite increased with the strength of the PAAm matrix. Regarding modulus anisotropy, the modulus of the composite was independent of the PAAm concentration when stretched parallel to the stem axis, while the modulus increased with the PAAm concentration when stretched perpendicularly.

**Figure 7.**
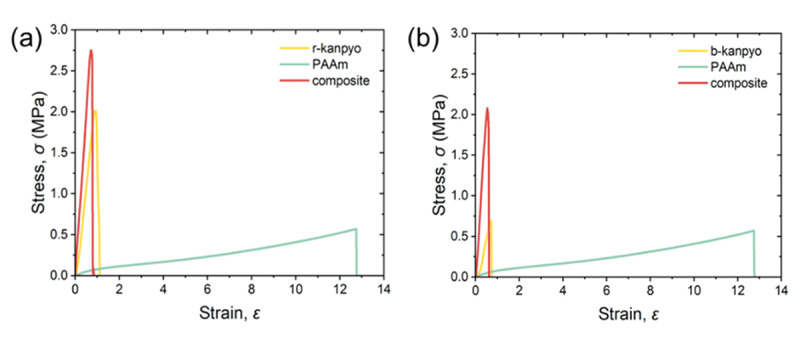
Tensile stress-strain curves of the (a) r-kanpyo/paam-6 and (b) b-kanpyo/paam-6 bio-composites and their parents.

Figure 7(a,b) shows the stress-strain curves of the r/b-kanpyo, PAAm-6, and r/b-kanpyo/PAAm-6 bio-composites stretched parallel to their long axes. The kanpyo/PAAm composites exhibited higher strength than the kanpyo alone, especially in the case of the b-kanpyo/PAAm composite. Analogous to the eringi composite, the integration of rigid kanpyo with a soft PAAm matrix can improve the strength of the kanpyo. The elastic modulus of the kanpyo composite was close to that of kanpyo alone, similar to when the eringi/PAAm composite was stretched parallel to the stem axis. In contrast to eringi, pre-boiling the kanpyo resulted in a decrease in the strength of the composites. This is likely because the fibrous strength of the kanpyo decreases upon boiling-induced hydration, while the amount of PAAm introduced into the composite remains unchanged by the boiling process.

To summarize the above, an increase in strength was observed in both eringi and kanpyo through complexation with PAAm. When stretched parallel to the fibers, the strain at break increased while maintaining the modulus of the fibrous agricultural products, resulting in an increase in fracture stress. When stretched perpendicular to the fibers, the modulus, strength, and strain at break were all improved. In this case, the modulus of the bio-composite was greater than the sum of those of the agricultural products and the matrix. The two types of toughening behavior in the bio-composite, depending on the stretching direction, are generally observed in long-fiber composites [7,18,30–32]. When a composite material of oriented fibers and matrix is stretched along the fiber direction, the composite fractures due to slippage (pulling out) or fracture of fibers. The soft matrix strongly bonded to the fibers does not contribute to stress of the composite so much but effectively suppresses slippage between the fibers. As a result, strength and stretchability of composites are improved while the modulus remained almost unchanged. When stretched perpendicular to the fibers, the composite fractures mainly due to interfacial debonding between the fiber and the matrix or fracture of the matrix. The presence of a matrix with robust bonding to the fibers strongly suppresses interfacial debonding, thereby suppressing fracture and resulting in an improvement in strength and elongation. It is noted that the PAAm matrices contribute significantly to the moduli of the composites stretched perpendicularly, even though modulus of the matrices alone is small. To explain it, localization of the deformation should be taken into account. In the bio-composites, a deformable soft matrix fills the gaps between a rigid fibrous phase. Upon global deformation of the composite perpendicular to the longitudinal axis, the rigid component undergoes minimal deformation, while the soft matrix experiences substantial local deformation. Therefore, even if the global strain of the composite remains low, the soft matrix is significantly stretched locally, generating high local stress and thereby markedly enhancing the modulus and strength of the composite under low global deformation.

For the effective toughening of the bio-composites, interface between the fibrous structures and PAAm matrices must be strongly bonded, ensuring their mechanical integration as mentioned. The hydroxyl groups in the biological tissue and the amide groups in polyacrylamide can both act as hydrogen bond donors and acceptors, suggesting the formation of hydrogen bonds between the two components [33,34]. These hydrogen bonds would efficiently transfer stress across the phase interface. We never observed separation between the biological tissue and the matrix during testing, which suggests an integration between the matrix and the biological tissue mediated by interfacial interactions.

**Figure 8.**
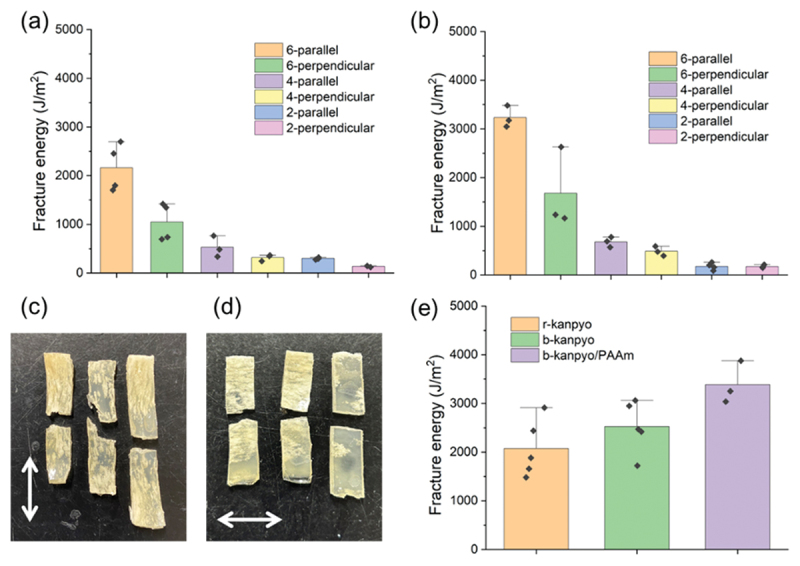
(a,b) Fracture energies of the eringi/PAAm-*C* bio-composites measured by the single edge-notch fracture tests, where parallel and perpendicular denote the direction of stretch; (a) r-eringi/PAAm-*C* composites, (b) b-eringi/PAAm-*C* composites. (c,d) the appearance of the r-eringi/paam-6 composite after the fracture test; (c) parallel, (d) perpendicular. White arrows indicate the stem axis. (e) Fracture energies of the r/b-kanpyo and the b-kanpyo/paam-6 bio-composite.

Single-edge notch tests were subsequently conducted to estimate the fracture toughness. Figure 8(a,b) exhibits the fracture energy of the eringi/PAAm-*C* bio-composites. The fracture energy of the composites increased with the PAAm concentration. This means that the fracture resistance of eringi was improved by incorporation with the flexible PAAm matrix, and the effect of toughness improvement becomes more obvious with its concentration. The b-eringi/PAAm composites exhibited higher fracture energy because of the denser PAAm inside the composites. When the specimen was stretched along the stem axis, corresponding to the crack propagation perpendicular to the stem axis, the fracture energy was notably higher. This directional effect was especially pronounced in the r-eringi/PAAm composites. Figure 8(c,d) shows appearance of the cracks propagated in the r-eringi/PAAm-6 composites. While the cracks smoothly propagated along the stem axis (Figure 8(d)), they did not propagate linearly in the direction perpendicular to the axis but sometimes branched (Figure 8(c)). This crack bifurcation is often observed in the fracture of anisotropic composites and has been found in our previous squid/PAAm bio-composite works [18,35]. The crack propagation is impeded by the rigid fibrous phase, resulting in vertical crack bifurcation to detour the fibers. This crack bifurcation effectively contributes to improve the fracture energy. Figure 8(e) shows the fracture energy of the r/b-kanpyo and b-kanpyo/PAAm-6 composites. The kanpyo itself exhibits high fracture energy of around 2000 J/m^2^, attributable to the robust fibers formed during its drying process. Upon compositing with PAAm, its fracture energy was further improved to approximately 3500 J/m^2^. In both eringi and kanpyo composites, the PAAm matrix, which serves as a stress transfer medium, can effectively disperse stress from the crack tip to the surrounding region. This stress dispersion significantly contributes to the enhancement of the fracture energy of the composites.

### 3.3. Eringi/gelatin bio-composites

We initially synthesized the bio-composites using synthetic polymer matrices. Replacing the synthetic polymer matrices with natural polymer matrices such as gelatin offers advantages in terms of environmental sustainability and biocompatibility, but this is challenging. To incorporate the PAAm matrix, the agricultural products are immersed in a monomer solution, allowing small monomers to diffuse into the tissue, followed by *in situ* polymerization of the monomers. Since the monomers are small molecules with a molecular weight on the order of 100, they readily diffuse into the tissue. In contrast, natural polymer matrices such as gelatin hydrogels are prepared not from monomers but from pre-formed long polymer chains. To successfully form a natural polymer matrix within fibrous bio-tissue, the average pore size of an agricultural product must be large enough to allow translational diffusion of biopolymers into the pores. Among the four types of agricultural products used in this study, only r-eringi has large pores on the order of 10 − 100 µm, which would allow translational diffusion of biopolymers into the products.

**Figure 9.**
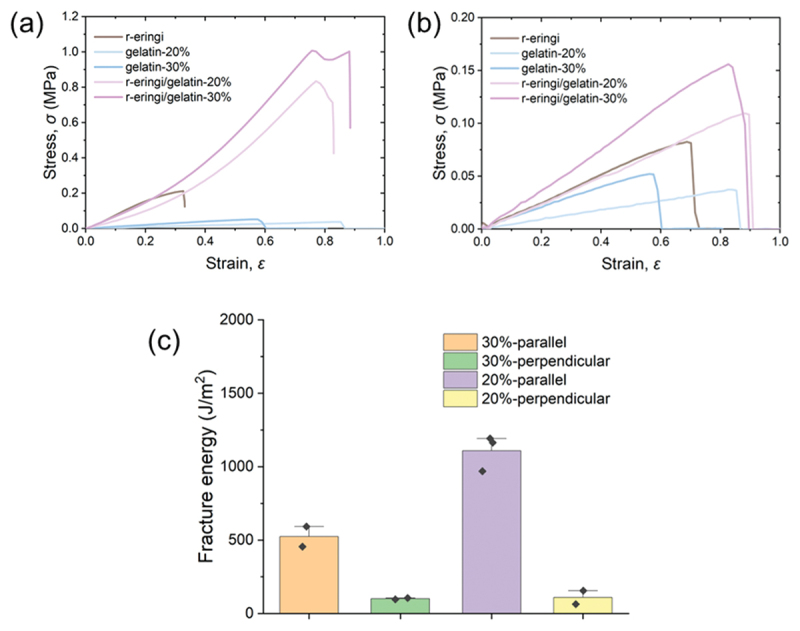
(a,b) Tensile stress-strain curves (a) parallel and (b) perpendicular to the long axis, and (c) fracture energies of the r-eringi/gelatin-*C* bio-composites.

The r-eringi was immersed in the hot aqueous gelatin solutions to allow sufficient diffusion of gelatin into the tissue, followed by cooling to fabricate the physical gelatin network and the eringi/gelatin bio-composites. Figure 9(a,b) shows the tensile test results of the eringi/gelatin-*C* bio-composites and their parents. The gelatin matrices were soft but not highly deformable. Similar to the eringi/PAAm composites, the eringi/gelatin composites stretched along the stem axis exhibited high strength. In contrast, very little toughening was seen when stretched perpendicular to the axis. This would be due to the above-mentioned toughening mechanism of the bio-composites depending on the stretching direction. When stretched parallel to the axis, the matrix serves to suppress slippage of the fibers. Since the matrix does not need to deform significantly for this effect, a matrix without large stretchability like gelatin can still contribute to toughening of the composites. On the other hand, when stretched perpendicular to the axis, the matrix bonded to the fibers undergoes large tensile deformation. In this case, the brittle gelatin matrices resulted in little strengthening of the bio-composites. Figure 9(c) shows the fracture test results of the eringi/gelatin bio-composites. Toughening similar to that observed in the eringi/PAAm composites was found, while the direction dependence of the fracture energy was more pronounced. This is related to the results of the tensile tests. Because the gelatin matrix has poor deformability, its intrinsic fracture energy would be relatively small. When a crack propagates in the composite along the fibers, the crack likely propagates inside the matrix between the two adjacent fibers. In this case, the matrix with low intrinsic fracture energy reduces the overall toughness of the composite.

Since the eringi/gelatin bio-composites are composed entirely of edible components, it is expected to be fully biodegradable, biocompatible, and also suitable as a food product. Their mechanical robustness and edibility suggest potential use as a novel food product with unique texture, such as a jerky substitute.

## 4. Conclusions

By introducing a polymer network into the internal structure of fibrous agricultural products with hierarchical alignment, we successfully fabricated robust bio-composites through a simple process. A key conceptual advantage of this approach is the formation of composites comprising aligned fibrous natural polymers without energy-consuming processing of natural polymers; decomposition, purification, and reassembly. While commercial agricultural products specialized for food were used in this study, future efforts may involve cultivating vegetables or mushrooms specifically optimized for fabrication of tough soft composites. This methodology might be different from the recent trend of creating bio-based materials using unused byproducts. However, the properties of materials made from such byproducts often remain moderate. If bio-composites made from a specific agricultural product exhibit truly exceptional properties, then producing that product in the field would be worthwhile.
